# Axonal plasticity underpins the functional recovery following surgical decompression in a rat model of cervical spondylotic myelopathy

**DOI:** 10.1186/s40478-016-0359-7

**Published:** 2016-08-23

**Authors:** Rana S. Dhillon, John Parker, Yasir A. Syed, Steve Edgley, Adam Young, James W. Fawcett, Nick D. Jeffery, Robin J. M. Franklin, Mark R. N. Kotter

**Affiliations:** 1 Department of Clinical Neurosciences, Anne McLaren Laboratory, Wellcome Trust-MRC Cambridge Stem Cell Institute, John van Geest Centre for Brain Repair, Academic Neurosurgery Unit, University of Cambridge, Cambridge Biomedical Campus, West Forvie Building, Forvie Site, Robinson Way, Cambridge, CB2 0SZ UK; 2 Wellcome Trust-Medical Research Council Cambridge Stem Cell Institute, University of Cambridge, Clifford Allbutt Building, Cambridge Biomedical Campus, Cambridge, CB2 0AH UK; 3 Department of Physiology, Development and Neuroscience, University of Cambridge, Downing Street, Cambridge, CB2 3DY UK; 4 Department of Clinical Neurosciences, John van Geest Centre for Brain Repair, University of Cambridge, E.D. Adrian Building, Forvie Site, Robinson Way, Cambridge, CB2 0PY UK; 5 College of Veterinary Medicine, Iowa State University, 1800 Christensen Drive, Ames, IA 50011-1134 USA

## Abstract

Cervical spondylotic myelopathy (CSM) is the most common spinal cord disorder and a major cause of disability in adults. Improvements following surgical decompression are limited and patients often remain severely disabled. Post mortem studies indicate that CSM is associated with profound axonal loss. However, our understanding of the pathophysiology of CSM remains limited.

To investigate the hypothesis that axonal plasticity plays a role in the recovery following surgical decompression, we adopted a novel preclinical model of mild to moderate CSM. Spinal cord compression resulted in significant locomotor deterioration, increased expression of the axonal injury marker APP, and loss of serotonergic fibres. Surgical decompression partially reversed the deficits and attenuated APP expression. Decompression was also associated with axonal sprouting, reflected in the restoration of serotonergic fibres and an increase of GAP43 expression. The re-expression of synaptophysin indicated the restoration of functional synapses following decompression. Promoting axonal plasticity may therefore be a therapeutic strategy for promoting neurological recovery in CSM.

**Electronic supplementary material:**

The online version of this article (doi:10.1186/s40478-016-0359-7) contains supplementary material, which is available to authorized users.

## Introduction

Cervical Spondylotic Myelopathy (CSM) is the most common spinal cord disorder and one of the major causes of disability in adulthood [35]. It is induced by degenerative changes occurring in the intervertebral discs triggering bony and ligamentous hypertrophy, which result in narrowing of the cervical canal. Ultimately, tethering and compression cause injury of the spinal cord and increasing neurological deficits [2, 3]. The cellular events leading from compression to myelopathic changes are less clear. Current evidence suggests that mechanical compromise results in ischemia and triggers axonal injury, inflammation, and apoptosis [2, 26, 42].

Although not without controversy [38], the accepted mainstay of treatment, especially for more severe cases of CSM, is surgical decompression [8]. A recent North American study of CSM confirmed that surgery can lead to significant improvements in CSM [16, 17]. Partial reversal of symptoms occurs after surgery over 3–12 months. This time frame implicates inherent regenerative or plastic changes within the spinal cord. Nevertheless, many patients remain disabled [29], and there are nonsurgical treatments available for improving outcome for CSM.

Human post mortem studies suggest that the early phase of CSM affects the lateral funiculi that contain the lateral corticospinal tracts, resulting in axonal loss [9, 25, 40]. This corresponds well with the observation that spastic gait, an upper motor neuron sign, is one of the earliest signs of CSM. Later stages affect the posterior columns and the central grey matter [25]. Furthermore, degeneration of anterior horn motor neurons at the level of spinal cord compression can result in a corresponding lower motor neuron deficiency. Morphologically these changes manifest themselves as degeneration of sensory axons and necrosis and cavitation of the central grey matter. As a result, loss of sensation, proprioception and sphincter control occurs. Another feature of CSM is myelin loss [25, 39, 51]. However, the extent to which there is primary demyelination as a result of oligodendrocyte pathology or myelin loss secondary to axonal degeneration remains unclear.

Existing models mimicking chronic cord compression in rats include insertion of expandable polymers [50], adjustable screws [33], and calcification-inducing polymers [28]. These result in loss of neurons, predominantly in the grey matter of the ventral horns at the lesion epicentre. Preferential neuronal loss has also been demonstrated in canine screw compression models [21] and twy-twy mouse models [1]. In addition, demyelination has been observed in some CSM models [28, 51], although myelin pallor or frank myelin loss is not generally present in expandable polymer animal models [30, 50].

Only two studies in the literature reported on the consequences of surgical decompression in animal models of CSM. The first study involved a canine model, in which cord compression was achieved by insertion of a posterior sublaminar Teflon washer and an anterior vertebral screw. Surgical decompression after 37–50 weeks resulted in neurological improvement [21]. However, the authors noted that basic histological findings showed little correlation with functional recovery; a cellular analysis was not conducted. More recently, Karadimas et al. demonstrated that surgical decompression leads to improved blood flow and can cause ischemia-reperfusion injury in a rat model. This elegant study provided evidence of oxidative damage in neurons at the previously compressed level, and that the damage can be attenuated by the sodium channel blocker riluzole [27].

On the basis of the extensive axonal degeneration seen in post mortem studies of CSM patients and in preclinical models, we theorised that axonal plasticity plays a role in the recovery following surgical decompression. To investigate this hypothesis, we developed a novel rat model of mild to moderate CSM in which transient cervical spinal cord compression is induced by sublaminar insertion of a water-absorbing polyurethane elastomer. Behavioral tests were conducted at weekly intervals for 10 weeks. The spinal cords were then surgically decompressed and the animals observed for further five weeks. At the end of the experiment, animals were sacrificed to study the cellular events that occur as a consequence of chronic cord compression and surgical decompression.

## Materials and methods

### Animal experiments

The present series of experiments involved a total of fifteen purpose-bred adult male Sprague-Dawley rats weighing 300–400 g (mean 342 g, Harlan Laboratories, Bicester, UK) that were randomly assigned to three experimental groups (*n* = 5 per group). 1) Control group receiving two sets of sham surgery without implantation of a polymer. 2) Compression group, in which a polymer was implanted and sham surgery was conducted at the time point of decompression. 3) Decompression group, in which a polymer was implanted and subsequently removed.

All experimental protocols were approved by the Home Office UK under the Animals Scientific Procedures Act 1986 incorporating Amendment Regulations SI 2012/3039. Animals were housed in environmentally enriched cages in groups of four, with 12 hour light cycles at room temperature of 22 degrees Celsius and fed ad libitum on Rat Diet 512 pellets (LabDiet, St Louis, USA).

### Surgical implantation of expandable polymers

Anaesthesia was induced using inhaled isofluorane gas (Sigma-Aldrich, Gillingham, UK) tritrated to 2.5 % and maintained at 2 %. A 3.5 cm midline skin incision was made from the inion to the vertebra prominens. Midline sharp dissection was performed to expose the deep paraspinal extensor musculature. This deep layer was preserved with subperiosteal dissection of the muscle attachments to the dorsal laminar surface. The cervical laminae were identified by counting from C1. The ligamentum flavum at C4/5 and C2/3 was excised to expose the underlying thecal sac. The implant was passed cranially from the C4/5 epidural space to the C2/3 laminar interspace, whilst minimizing dorsoventral displacement of the thecal sac and secured on the dorsal aspect of the laminae at C3 and C4. Layered closure was performed and sutures removed on day 7 post operatively.

#### Compression material

A water-absorbing polyurethane elastomer (Aquaprene G, Sanyo Chemical Industries, Kyoto, Japan) was used. This was based on the polymer of an established pre-clinical model which reached a volume of 230 % over 24 h and remained stable for 16 days thereafter [30]. Sections measuring 0.70 mm (ventrodorsal) × 3 mm (mediolateral) × 5 mm (rostrocaudal) were cut and sterilised using ethylene oxide to prevent expansion from steam sterilisation.

#### Optimisation of the compression model

In preliminary experiments, implants of varying size and cervical level were trialed. Implants measuring 0.60 mm were found to produce minimal or no deficits and implants measuring up to 0.85 mm were found to induce severe neurological deficits. Implants of size 0.7 mm produced a moderate neurological deficit whose cadence most closely resembled the human condition. Cervical levels C5-7 were also trialed however these resulted in severe deficits, which prevented longer term investigation of the animals.

#### Sham surgery

Animals assigned to the sham surgery group underwent the same preparation, exposure and closure as the compression group. The implant was passed into the dorsal epidural space and then removed.

#### Surgical decompression

Animals assigned to the decompression group underwent the same preparation, exposure and closure as the compression group. The implant was removed through the caudal laminar interspace avoiding sudden ventrodorsal angulation. A single level bilateral laminectomy of the inferior level, namely C4, was performed.

#### Neurobehavioural assessments

Animals were subjected to the following neurobehavioural tests.

*The Basso, Beattie, and Bresnahan (BBB) score* assessed joint movement, weight bearing, paw placement, stepping, forelimb-hindlimb co-ordination, paw rotation at contact and lift off phases of gait, toe clearance, tail elevation, and trunk stability [4] in an open field measuring 95 × 45 cm. Four minute videos were recorded of each animal at each time-point and scored in random order to blind the assessor to time-points.

*Forepaw and hindpaw slips* were assessed using a 100 × 10 cm walkway with a wire floor consisting of 1.5 × 1.5 cm squares of wire thickness 1.6 mm, adapted from Bradbury and colleagues [7]. Videos were recorded of each animal at each time-point completing the walk 3 times and scored in random order. A slip was recorded if the paw fell below the plane of the grid, and the average number of slips after three walks calculated to give a score.

*Stride length and width* were assessed by inking the forepaws and allowing the rat to ambulate 3 times along a 1 m × 10 cm wooden walkway, adapted from Kunkel-Bagden et al. [31]. Stride width was taken as the distance between the central pads of the forepaws measured over 3 steps and averaged. Stride length was measured as the distance between two consecutive steps, measured over 3 steps and then averaged.

### Electrophysiology

Sensory evoked potentials were performed as terminal procedures on 2 rats that were randomly selected from each group. Anaesthesia was induced using 5 mg/kg of xylazine hydrochloride (Bayer, Cambridge, UK) and 100 mg/kg of ketamine hydrochloride (Pfizer, Kent, UK) injected intra-peritoneally. Three contact sites were exposed; the tibial nerve in the left posterior thigh, the gracile nucleus in the brainstem at the level of the obex, accessed through a posterior suboccipital approach, and the primary sensory cortex through a burrhole 1.5 mm anterolateral to bregma.

Stimuli were delivered to the tibial nerve via a cuff electrode with the nerve in contact with 2 silver wires 5 mm apart. Square wave pulses of 0.2 ms duration (best for stimulating smaller fibres), at 2 Hz, were delivered using an A-M Systems Isolated Pulse Stimulator Model 2100 (A-M Systems, Se-quim, USA). Signals were sampled simultaneously from the ipsilateral gracile nucleus and from the contralateral sensorimotor cortex via insulated silver wire electrodes. Nerve action potentials were amplified 2000 times (Neurolog NL824) filtered (5Hz to 5 kHz) and any mains contamnination removed using a Humbug device (Digitimer UK). Data were sampled at 25 kHz using via a Power 1401 acquisition system and Spike 2 data analysis software (Cambridge Electronic Design, Cambridge, UK).

Stimulus intensity was determined relative to the threshold of the fastest conducting fibres in the tibial nerve. This was the lowest current required to activate a compound action potential larger than the background noise and was typically 20–40 microamps. To assess responses in the gracile and sensorimotor cortex the nerve was then activated at four times that threshold current (T). For each measure an average of 100 SEP responses was taken, responses evoked from the tibial nerve and recorded at the gracile nucleus were recorded as spinal latencies, the fastest of which would be direct (no synaptic relays). Responses recorded at the sensory cortex were cortical latencies, comprising spinal latencies, plus synaptic delays at the gracile nucleus and sensory thalamus. Latencies were converted to velocities. Amplitudes of the spinal and cortical potentials were also recorded.

### Tissue processing

All histology was performed at the end of the experiment, after 10 weeks of compression and 5 weeks after decompression. Animals were administered 50 mg/kg of sodium pentobarbital intra-peritoneally (Sigma-Aldrich, Gillingham, UK). The left ventricle was catheterised via a midline sternotomy. Heparinised saline 10,000U/1000 ml (Sigma-Aldrich, Gillingham, UK) was perfused at 140 mmHg for 3 min followed by 4 % paraformaldehyde (Sigma-Aldrich, Gillingham, UK) at 140 mmHg for 7 min. The neural axis was removed en bloc and post-fixed in 4 % PFA overnight. Tissues were left in 30 % sucrose overnight for cryoprotection then frozen in Tissue-Tek® OCT matrix (TissueTek, Sakura Finetek, Thatcham, UK) at −70 °C. Longitudinal sections of cervical cord of 12 μm thickness spanning 5 mm above and below the lesion were made on a cryostat (OTF 5030, Bright Instruments, Huntingdon, UK), mounted onto polylysine coated slides (Menzel-Glaser, Thermo Fisher Scientific, Waltham, USA) and stored at −20 °C.

### Immunohistochemistry

Frozen sections were thawed at room temperature. Where indicated, heat-induced epitope retrieval was performed by incubating sections in citrate buffered antigen retrieval solution (Dako, Ely, UK) at 90° for 10 min. Sections were blocked in 5 % normal goat serum (ab7481, Abcam, Cambridge, UK) for 2 h. Primary antibodies were applied and incubated overnight at 4 °C. The following primary antibodies and concentrations were used; rabbit anti-amyloid precursor protein (PAD CT695, Life Technologies, Manchester, UK), rat anti-serotonin at 1:1000 (ab6336, Abcam, Cambridge, UK), rabbit anti-synaptophysin at 1:1000 (ab32127, Abcam, Cambridge, UK), rabbit anti-GAP43 at 1:1000 (ab11136, Abcam, Cambridge, UK), mouse anti-glial fibrillary acidic protein (GFAP) at 1:500 (ab7260, Abcam, Cambridge, UK), goat anti-Iba1 at 1:500 (ab5076, Abcam, Cambridge, UK), rabbit anti-Olig2 at 1:500 (P21954, LifeTechnologies, Manchester, UK) mouse anti-APC (aka anti-CC1) at 1:300 (OP80, Merck Millipore, Nottingham, UK), Rabbit, and anti-Caspase 3 at 1:500 (ab13847, Abcam, UK).

Goat secondary antibodies diluted to 1:500 of emission wavelength 488, 555 and 647 nm (Alexa Fluor Life Technologies, Manchester, UK) were used, except for the goat anti-Iba1 primary for which a donkey 555 secondary antibody was used (Alexa Fluor Life Technologies, Manchester, UK). Sections were counterstained with 4',6-diamidino-2-phenylindole (DAPI), then mounted and coverslipped.

Negative controls were performed by excluding the primary antibody. Cervical cord cranial to the lesion was used for positive controls for stains assessing axonal sprouting. Caudal cerebellar peduncle lesions from our laboratory were used as positive controls for stains assessing myelination and the immune response. Fluoromyelin at 1:300 (F34651, Life Technologies, Manchester, UK) for 20 min was used as a myelin stain. BDA staining was performed by first blocking endogenous peroxidases in 0.3 % H202 and 10 % methanol in PBS for 20 min. Sections were then incubated in avidin/biotinylated enzyme complex (ABC) (PK-4000, Vector laboratories, Burlingame, CA, USA) for 30 min. The biotinylated signal was then amplified by incubating sections in biotinyl tyramide for 10 min (SAT700, Perkin Elmer, Waltham, MA, USA). Sections were then incubated in 1:500 avidin overnight at 4 °C. An unamplified control with ABC and an amplified control without ABC were also performed.

### Image processing

Tissues were visualized at room temperature with a LSM 700 confocal laser scanning microscope (Zeiss, Cambridge, UK) and digitalized using Zen 2009 software (Zeiss, Cambridge, UK) then saved in laser scanning microscope format (.lsm). Image analysis was performed using Image J software (US National Institutes of Health, Bethseda, Maryland, USA).

### Immunohistochemical analysis

Tissue samples were analyzed blinded from each group (ie control, compression or decompressed) at three different regions: 2 mm cranial, 2 mm caudal, and at the site of compression (lesion).

Cell bodies of neurons and APP plaques and synaptophysin were analyzed in the grey matter. Oligodendrocyte lineage cells, astrocytes and microglia were counted in both grey and white matter with results presented in the white matter.

#### Quantification of immunofluorescent cells

APP+ cells (and plaques), Caspase3+, Iba-1+, Olig2+ (and Olig2+/CC1+), and GFAP+ cells were all counted manually on 5 random visual fields per region (cranial, lesion, caudal). Using an Olympus IX71 light microscope with an Olympus U-PFL-T fluorescent lamp and a TH4-200 bright-field lamp at 20x magnification, cells that stained for markers of interest were manually counted using a hand held tally counter. The obtained cell counts were averaged to give a representative count for each specific region.

#### Quantification of axonal markers

APP and GAP43 immunodensities were quantified in the white matter using ImageJ software (US National Institutes of Health, Bethseda, Maryland, USA). Images were converted to grey scale and thresholded. APP and GAP43 was quantified as the fraction of the area above the threshold within the region of interest. Results were recorded as ‘% of positive tissue’.

Synaptophysin in the grey matter was quantified as density of staining relative to white matter. Fluoromyelin was quantified in the same manner as synaptophysin, except that white matter immunodensities were normalized to grey matter immunodensities. For synaptophysin and fluoromyelin the process was repeated 16 times per image and averaged. The value was recorded as ‘Arbitrary units’.

#### Quantification of axonal sprouting

Sprouting of 5HT-labelled raphespinal axons was based on a modified version of a published technique (Zhao et al. 2013). Images were examined at 10x magnification and divided into 2 mm rostral to the lesion, the lesion itself, and 2 mm caudal to the lesion. Each section was then analysed in 0.5 mm increments. The number of branches coming off an axon were counted, and this was divided by the total number of labelled axons in that region to give a count of sprouts per axon. In addition, 5HT immunodensity was quantified as per Lee and colleagues (Lee et al. 2010) and described above.

### Statistical analysis

Results were analyzed using GraphPad Prism Software Statistics GradPack 18. Immunohistochemistry quantifications were assessed using a parametric one-way analysis of variance (ANOVA). When the null hypothesis was rejected using one-way ANOVA, we then applied a Fisher’s LSD post test. Functional quantifications were assessed using two-way ANOVA followed by a Dunnett’s multiple comparison post-hoc test by defining a control mean to determine which of the groups had a significant difference. We pre-determined a significance level (alpha) of 0.05.

## Results

### Chronic compression impaired motor function that was partly reversed by decompression

A pre-clinical model of CSM was established by surgically inserting an expandable polymer in the dorsal cervical epidural space of C3/4. The size of the polymer was chosen to cause moderate neurological signs (see Methods section: optimisation of compression model). Following implantation, the polymer expanded resulting in gradual compression of the spinal cord. Controls received sham surgeries during which polymers were transiently inserted into the sub-laminar space, but removed before surgical closure. After a course of 10 weeks of compression, a laminectomy was conducted and the implants were removed to decompress the spinal cord (decompression group). Sham surgery was conducted on compression only animals and controls.

**Fig. 1:**
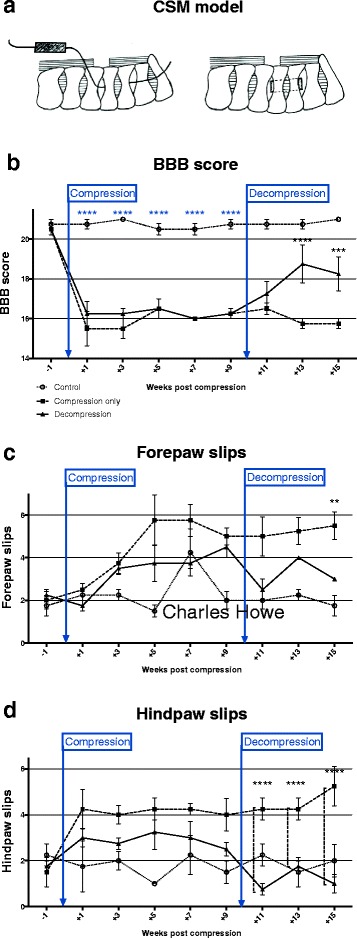
**a** Chronic cord compression was induced by surgical implantation of an expandable polymer underneath the posterior arches of C3/4. Sham surgery was conducted on controls (each experimental group *n* = 5). **b** Locomotor behaviour was assessed using open-field Basso Beattie Bresnahan (BBB). Spinal cord compression resulted in significant neurological deterioration within 1 week (****p < 0.0001). After 10 weeks, a laminectomy was performed and the implants removed. In the decompressed group, scores improved significantly three weeks after surgery (****p < 0.0001). Induction of SC compression also increased the number (**c**) forepaw and (**d**) hindpaw slips of rats placed on a grid as compared to controls. On the other hand, a significant reduction of forepaw and hindpaw slips was detected following surgical decompression (**p < 0.01, ****p < 0.0001)

Animals underwent neurobehavioural testing over a period of 15 weeks. Subsequently animals were sacrificed and the tissues analysed. As the main purpose of the study was to investigate basis of functional recovery, it was important to first establish functional changes occurring in the model and following decompression. Neurobehavioural assessments included the Basso Beattie and Bresnahan (BBB) locomotor behavior score [4], and assessment of forepaw and hindpaw slips when rats were placed on a 16 gauge wire grid (Fig. 1).

One week after compressive surgery, neurological deterioration was detected in compressed animals as compared to controls. This was reflected in a significant drop in BBB scores (mean difference at week + 1 = −5.25; post-hoc Dunnett’s multiple comparison test following two-way ANOVA: *p* < 0.0001). Surgical decompression after 10 weeks led to gradual functional improvement, which reached statistical significance after three weeks (mean difference week + 13 = −2.88, two-way ANOVA: *p* < 0.0001, post-hoc Fisher’s LSD test: *p* < 0.0001–0.001). However, the functional and electrophysiological recovery (Additional file 1: Figure S1) remained incomplete.

Compression also induced an increase in the number of forepaw and hindpaw slips in rats placed on a wire grid (mean difference at week + 5 = 2.5). Conversely, a decrease in forepaw slips (mean difference at week + 15 = −2.5) was noted after decompressive surgery (post-hoc Tukey’s multiple comparison test: *p* < 0.01). A similar result was seen for hindpaw slips, which decreased in decompressed rats by 1.38 compared to compressed rats (post-hoc Tukey’s multiple comparison test: *p* < 0.0001).

### Apoptosis induced by chronic cord compression is attenuated by decompression

**Fig. 2:**
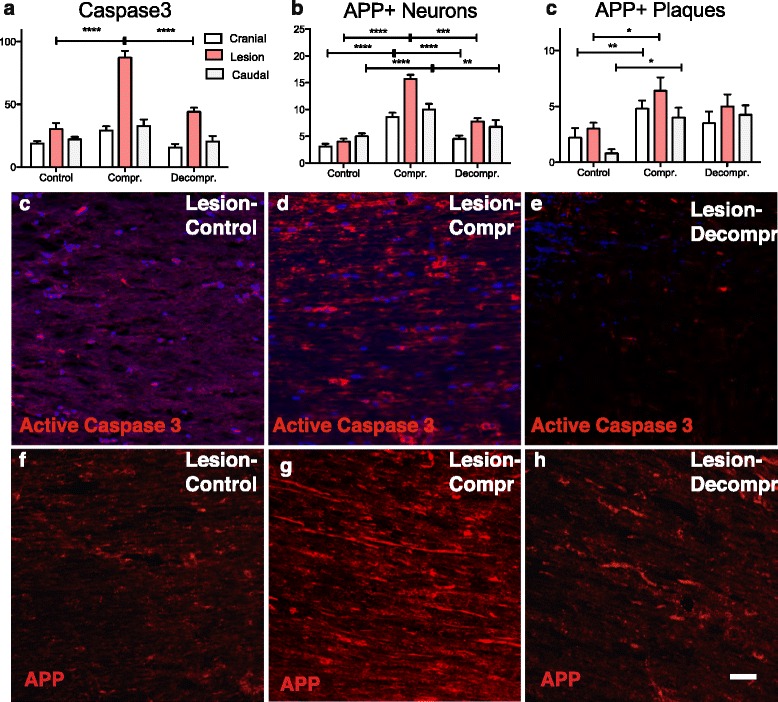
To assess the consequences of compression and decompression on cell apoptosis, sections were stained for Caspase-3. **a**-**e** Quantification of Caspase3-positive cells cranial, caudal and at the lesions sites demonstrated increased levels of apoptosis as a result of chronic cord compression at the lesion site (****p < 0.0001). Conversely, the number of apoptotic cells decreased significantly approaching normal levels following surgical decompression (****p < 0.0001). To assess neuronal pathology, sections were stained for APP. Quantification of APP+ axons in white matter tracts demonstrated a significantly increased APP expression as a consequence of compression (*p < 0.05, ****p < 0.0001), which subsided after decompression (***p < 0.001, ****p < 0.0001). Similarly, the number of APP+ but morphologically intact neurons in the grey matter increased as a result of compression cranial, caudal and at the lesions site (****p < 0.0001), and decreased following decompression to levels observed in controls (**p < 0.01, ***p < 0.001, ****p < 0.0001). In addition, the number of APP+ plaques in the grey matter significantly increased after decompression (*p < 0.05, **p < 0.001), but failed to decrease following decompression, indicating a sub-population of cells that was irreversibly damaged. Scale bar: **c**-**h** = 100 μm

Cellular changes associated with compression and decompression were studied by immunohistochemistry on sections cranial, caudal, and at the lesion site. Quantification of caspase3-positive cells demonstrated a sharp increase in apoptosis associated with chronic cord compression. The increase of caspase3-positive cells occurred predominantly at the lesion site. Apoptotic cells were found throughout the white and grey matter of the spinal cord. After surgical decompression, the density of caspase3-positive cells significantly reduced (Fig. 2; mean values at site of compression: control = 30.25, compression = 87.25, decompression = 44.00).

### Chronic compression leads to accumulation of amyloid precursor protein in neurons

Our hypothesis was that CSM involves axonal injury and may therefore be amenable to axonal plasticity. Amyloid precursor protein is a membrane spanning glycoprotein that is normally found in neurons and was first shown in the traumatic brain literature to be a marker for damaged axons [43]. Positive staining for APP in axons is thought to represent accumulation of the protein due to disruption of axoplasmic flow [44]. In the present study APP immunostaining was used as a marker of axonal and neuronal injury. We first quantified intensity of APP immunoreactivity in white matter tracts, which showed a marked increase at the site of compression, indicative of widespread axonal injury (Fig. 2; mean values at lesion site: control = 2.93, compression = 9.30, decompression = 1.23). Moreover, we assessed the number of APP-positive neurons with intact morphology in the grey matter, which are thought represent a potentially reversible stage of injury. Significantly increased APP expression in neuronal cell bodies was found in compressed rats above, below, and at the lesion centre (mean values at lesion site: control = 4.00, compression = 15.70, decompression = 7.75). This further supports the notion that chronic cord compression induces neuronal stress (Fig. 2). Finally, we also quantified the number of APP-positive plaques [13]. Cord compression induced a significant increase of APP plaques in the grey matter (mean values at lesion site: control = 3.00, compression = 6.40, decompression = 5.00; data not shown).

### Surgical decompression reverses APP accumulation

Following surgical decompression, a pronounced decrease of APP immunoreactivity in white matter tracts and APP-positive neurons in grey matter was detected above, below, and at the lesion site (Fig. 2). The levels of APP expression in white matter tracts and the number of APP-positive neurons after surgery approached levels of non-injured controls. In contrast, the number of APP-positive plaques did not change.

### Chronic compression results in degeneration of serotonergic axons and loss of synapses

**Fig. 3:**
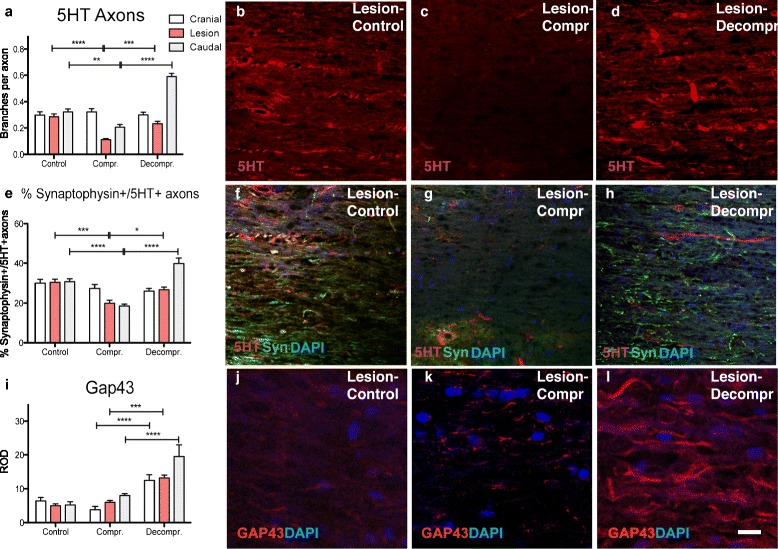
To further explore the effects of spinal cord compression and decompression serotonergic axons were visualised by immunohistochemical staining for 5HT. **a**-**d** Compression resulted in a significant decrease of 5HT positive axons in white matter tracts above and at the site of cord compression (**p < 0.01, ****p < 0.0001. On the other hand, the presence of 5HT fibres increased significantly at and below of the area of cord compression following surgical decompression. **e**-**h** Quantification of 5HT+/synaptophysin + axons in the grey matter demonstrated a decrease in functional serotonergic innervation at and below the lesion site (**p < 0.01, ****p < 0.0001). Following decompression, a significant increase of 5HT+/synaptophysin + axons was detected (***p < 0.001, ****p < 0.0001), which points to the presence of axonal sprouting. **j**-**l** Axonal sprouting was further confirmed by immunohistochemistry for GAP43, which indicated an increase of GAP44+ axons below the lesion site following decompression (***p < 0.001, ****p < 0.0001). Scale bar: **b**-**d**, **f**-**h** = 100 μm; **j**-**l** = 30 μm

To further assess neuronal damage, serotonergic axons of the descending raphespinal tract were investigated in the spinal cord. At the centre of compression, a significant loss 5HT-positive axons occurred (Fig. 3; mean values at lesion site: control = 0.36, compression = 0.08, decompression = 0.30). To further assess the functional connections, immunohistochemical staining for synaptophysin was conducted. Compression groups had lower levels of synaptophysin immunostaining compared to control, suggesting that chronic cord compression results in a loss of synapses at the site of compression (data not shown). Furthermore, compression reduced the number of HT5+/synaptophysin + axons, demonstrating loss of descending serotonergic input (Fig. 3; mean values at lesion site: control = 45.4, compression = 15.60, decompression = 47.75).

### Surgical decompression induces axonal sprouting and formation of new synapses

Previous studies of acute SCI indicate that serotonergic axons may have an increased propensity to sprout in response to injury [23] and accordingly, could serve as a potentially sensitive assay of axonal plasticity. We found that serotonergic fibre number at the lesion site increased significantly following surgical decompression. Regenerative sprouting of serotonergic fibres was most pronounced caudal to the lesion, where the decompressed group displayed significantly more serotonergic axons compared to all other groups (Fig. 3a; mean values below previous compression: control = 0.380, compression = 0.220, decompression = 0.638).

However, were the sprouting raphespinal fibres able to form functional connections and so contribute to the functional improvement seen following decompression? This is difficult to demonstrate definitively, but one line of evidence that has been used as a correlate of functional synapses is the co-localisation of synaptophysin with HT5 [6]. Quantification of synaptophysin-positive serotonergic fibres demonstrated a significant reduction in HT5+/synaptophysin + axons in chronically compressed spinal cords at and caudal to the lesion (Fig. 3e; mean values at lesion site: control = 0.285, compression = 0.112, decompression = 0.233). After decompression the proportion of HT5+/synaptophysin + axons increased significantly, reaching levels seen in controls at the lesion site, and increasing above control levels caudal to the lesion. Sprouting serotonergic axons were therefore likely to be forming synapses.

Another way to assay plasticity is to test for surrogate markers such as GAP-43. This membrane bound protein is expressed in extending axons and its expression likely represents a high-growth state [47]. Following decompression, GAP-43 expression was strongly induced above, below, and at the lesion itself (Fig. 3j; mean values at lesion site: control = 5.0, compression = 6.0, decompression = 13.2).

### Effects of compression and decompression on microglia, astrocytes and myelin

**Fig. 4:**
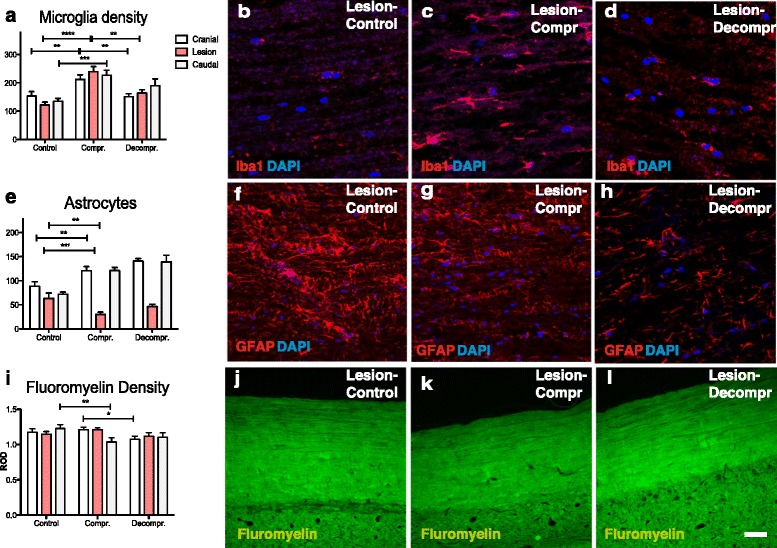
**a**-**d** The microglial response was assessed by staining for Iba1. Compression induced a significant accumulation of Iba1+ cells above, below, and at the site of cord compression (**p < 0.01, ***p < 0.001, ****p < 0.0001). Following decompression, Iba + microglia reduced above and at the site of compression (**p < 0.01). **e**-**h**) Interestingly, compression resulted in a marked loss of GFAP-positive astrocytes at the level of the lesion (**p < 0.01). Conversely, a marked astrocytosis was detected above and below the site of compression (**p < 0.01, ***p < 0.001). The absence of astrocytes at the area of compression and the reactive astrocytosis above and below persisted following decompression. **i**-**l** Visualization of myelin with fluorescent dies (Fluoromyelin) failed to demonstrate frank demyelination. Scale bar: **b**-**d** = 0 μm; **f**-**h**, **j**-**l** = 100 μm

To characterize the innate immune response, Iba-1-positive microglia were quantified. Following compression, a significant increase in the number of Iba-1-positive cells occurred, above, below and at the site of maximal compression. Once the pressure was relieved, the inflammatory response subsided and the number of microglia reduced approaching background levels in all areas investigated (Fig. 4a-d; mean values at lesion site: control = 121.6, compression = 239.4, decompression = 164.0).

We next quantified the reaction of astrocytes to compression and decompression by staining for GFAP (Fig. 4e-h). We found a marked loss of GFAP-positive astrocytes at the site of compression. This was in contrast to the findings above and below of the lesion, where a significant increase in GFAP immunoreactivity occurred. Decompression had no detectable effects on GFAP staining: GFAP levels at the site of compression remained low whereas increased levels of astrocytes persisted above and below the lesion (mean values at lesion site: control = 63.4, compression = 30.2, decompression = 46.5).

Finally, we stained membranous components in the spinal cords using a fluorescent dye (fluoromyelin). We observed a decrease in fluorescence in the compressed group compared to controls caudal to the lesion. Moreover, fluorescence intensity in the decompressed group above the lesion was decreased compared to compressed animals (Fig. 4i-l, mean values at lesion site: control = 1.146, compression = 1.208, decompression = 1.116).

## Discussion

### Recapitulation of clinical CSM

We propose a preclinical model of CSM that resembles a moderate clinical phenotype in human CSM patients. Histological analysis demonstrated that chronic cord compression compromised axons and damaged neurons, and resulted in loss of axonal and synaptic integrity. These findings faithfully reproduce findings of human autopsy studies of CSM patients, which detected axonal loss [25] and an increase in APP immunoreactivity at the compression epicentre [51]. They are also consistent with results of other expandable polymer rat models of CSM that found a correlation between compression and functional impairment [28, 30]. In the present model, hind-forelimb coordination assessed by the BBB score and quantification of hindpaw slips proved to be more consistent for monitoring neurological deficits than the quantification of forepaw slips. A potential explanation is the discrepancy in the extent of innervation to the forepaws as compared to the hindpaws.

The hypothesis of the present study was that decompression would trigger and enable a regenerative response in axons. Our results demonstrated that surgical decompression is able to partially restore function. This fits well with observations in human patients, where improvements following surgery have been reported independent of disease severity [16, 17].

Surgical decompression increases spinal cord blood flow and results in changes in the metabolic milieu. These changes by themselves may result in immediate improvements of cellular and axonal functions. However, in the present model functional recovery did not immediately follow decompression but occurred gradually over a three-week period. This resembles the expected time frame of axonal plasticity. Similarly, the benefits from surgical decompression in humans do not manifest themselves immediately. Systematic studies of CSM patients that were decompressed indicate that improvements occur over several months and can be between 3 and 12 months post-operatively [17]. Follow up investigations after 24 months indicate that these improvements often persist, and that surgery therefore can lead to continued benefits for CSM patients [16]. This also suggests that surgical decompression is able to halt the tissue destruction caused by chronic cord compression.

Similar to human CSM, the present model is based on chronic compression of the spinal cord. However, expansion of the implant after implantation is unlikely to fully reflect the slowly progressive nature of human disease. Moreover, compression in the present model is only mediated from posterior, whereas in human CSM it can occur selectively anterior, posterior, or circumferentially. The method of decompression in the present model is consistent with a posterior decompression in human patients, which has been shown to be comparable to anterior decompression in terms of post-operative outcome [15, 32].

#### Chronic cord compression causes axonal degeneration and neuronal loss

Quantification of Caspase3-positive cells demonstrated increased apoptosis at the level of compression. In addition, our findings indicated substantial axonal and neuronal injury: APP intensity in white matter tracts was markedly increased at the site of compression. The accumulation of APP in axons suggests that there was a degree of cytoskeletal breakdown in CSM. Amyloid precursor protein is transported by fast axonal trafficking and accumulates at detectable levels at defects in the cytoskeleton [43]. Whether this is due to direct mechanical trauma, ischaemia, or some other mechanism remains to be established. One possible explanation for the observed APP accumulation is that anterograde transport may be affected more than retrograde transport.

Cord compression also induced accumulation of APP in neurons in the central grey matter above, below and at the site of compression. APP accumulation in morphologically intact neurons appeared to be reversed following decompression, indicative of sublethal damage. However, the number of APP-positive plaques was not reversible.

Although its role is only partially understood, descending serotonergic input to the spinal card by the raphespinal tract has implicated in the control of the central pattern generator (for an excellent review see [19]). Quantification of descending serotonergic fibres as a relevant subset of axons in the spinal cord detected a profound loss of serotonergic axons at the epicentre as a result of spinal cord compression. The implied loss of connectivity was directly reflected by the loss of synapses, as indicated by the loss of synaptophysin staining above and at the level of compression.

Taken together, our findings demonstrate that chronic cord compression causes a significant neuronal phenotype with profound axonal injury. This fits well with observations in human post mortem studies of CSM patients demonstrating a prominent loss of axonal and neuronal elements.

To our knowledge, the present study is the first to systematically study the cellular and molecular consequences of surgical decompression in a model of CSM. As discussed above, decompression resulted in a gradual improvement of function, within a time frame that is suggestive of an underlying regenerative/plastic response. Assessment of Caspase3 immunohistochemistry demonstrated a significant reduction in apoptosis with levels reaching base line after 5 weeks following decompression. Surgical decompression therefore terminated the ongoing cell loss via apoptosis. Similarly, the reduction in APP immunoreactivity suggests that surgical decompression is able to halt the cellular damage caused by chronic cord compression. This is consistent with clinical data demonstrating persistent improvements following surgical decompression [16]. However, long term follow up studies are required to rule out that prolonged compression of the spinal cord does not trigger a slowly progressive neurological decline, as is often seen in other neurodegenerative conditions [48].

### Surgical decompression enables axonal plasticity and promotes the formation of synapses

Assessment of 5HT immunohistochemistry following decompression demonstrated a marked increase of serotonergic fibres, above, below and at the site of previous compression. This increase is likely mediated by a localized sprouting response of serotonergic fibres. The notion that the observed increase of serotonergic fibres is a result of axonal regeneration is supported by the concomitant presence of Gap43, a protein that is expressed during high-growth states of axons [5, 7], and which was detected above, below, and at the site of previous compression. In addition, we found that following decompression, synaptophysin was re-expressed. Synaptophysin expression is thought to represent functional synapses [12, 20, 41]. Taken together, these data demonstrate that surgical decompression triggers a regenerative response in axons that leads to the establishment of new, functional connections.

### Glial reactions to surgical decompression

We observed a marked microglial response to compression which extended to the adjacent tissue above and below the epicentre. Following decompression, microglia activation was reduced. The fact that microglia are activated in CSM was expected and corresponds well with findings of other CSM models [22, 24, 28, 51] and post mortem studies of CSM [51]. Similarly, PET studies conducted in clinical patients confirmed that cord compression in CSM is associated with inflammation [18].

In the present study, we did not find evidence of gross myelin destruction in our model. Compression resulted in decreased fluoromyelin below the lesion site, whereas decompression led to a decrease above the compression site. These findings are difficult to interpret and warrant further investigation with more sensitive techniques. Another striking finding was the loss of GFAP staining at the epicenter of compression with a concomitant increase of GFAP+ cells above and below the are of compression. Interestingly, astrocytes were unable to recover after decompression and thus the loss of GFAP immunohistochemistry persisted in the present study. Astrocytes are known to play an important role in the formation of the glial scar, the maintenance of the BBB, and regenerative responses, including remyelination [46]. Experimental depletion of astrocytes in various injury paradigms is associated with increased spread and persistence of inflammatory cells, more persistent loss of BBB function, and increased tissue damage [11, 14, 36, 49]. It is possible that the reduced astrocyte activity may have facilitated axonal sprouting and attenuated demyelination, as seen following experimental modulation of astrocytes in experimental models of SCI [34]. Unfortunately, there are very few manuscripts investigating astrocytosis in human CSM and quantitative data remains wanting [45].

There are a number of limitations to the present study. Although our study demonstrated functional improvement based on behavioural tests (electrophysiological findings, Additional file 1: Figure S1) and concomitant histological evidence of axonal plasticity, a causal relationship between the two has not been formally demonstrated. Nevertheless, it is likely that the observed functional changes can be attributed to neuronal and axonal changes: 1) the loss of function observed in our model correlated well with the damage of neuronal elements. 2) The functional recovery observed after decompression, was notably associated with regenerative growth of axons. 3) Importantly, re-expression of synaptophysin is considered a surrogate marker of functional synapses as it is only expressed when active synapses are formed. 4) The time lag with which the improvements were observed, fits well with the time frame that is expected for successful axonal plasticity. However, the present study did not assess plasticity at other levels such as the cortex, subcortical nuclei and lumbar central pattern generators, which may also contribute to the improvements observed after surgical decompression [10]. Further studies are needed to assess alternative mechanisms of neural plasticity and conduct a more detailed and sensitive analysis of motor and sensory functions, specifically also including forepaw functions, which are clinically of significant relevance.

## Conclusions

CSM is a common and debilitating disease that affects a large number of individuals. The economic burden of the disease has not been calculated yet but is likely to be large. With continued aging of the populations in the industrial world, the incidence and prevalence of CSM is expected to increase. Concentrated efforts are therefore required to develop therapeutic options for promoting functional recovery from CSM.

The present model allows several clinically relevant and important questions to be addressed. For example, age has recently been recognized as an important determinant of functional outcome following surgery for CSM [37]. But the question how age affects the recovery from chronic compression remains unclear. Further questions include, how the duration of compression influences symptom severity and the ability to recover from CSM? Does changing the severity of cord compression alter the cellular and molecular response, how does it affect recovery? Importantly, our model will also permit to gain further insights in the pathophysiology of the disease and investigate treatment options for ameliorating the neurological deficits in CSM. Our results indicate that promoting axonal plasticity is one potential strategy to improve neurological function in individuals affected by CSM.

## Additional file

Additional file 1: Figure S1. Functional changes in the present model were reflected in sensory evoked potentials. For technical reasons only two rats were recorded in each group. Speed and amplitude of impulses transmitted from the tibial nerve in the lower limb to recording sites in the gracile nucleus of the lower brainstem (spinal) and the sensory cortex (cortical) were measured. Compression resulted in a decreased amplitudes of cortical and spinal waveforms. After decompressive surgery these increased but did not reach baseline amplitudes. (PDF 117 kb)
