# Supplementary figures and images for: Axonal plasticity underpins the functional recovery following surgical decompression in a rat model of cervical spondylotic myelopathy
Source: Acta Neuropathol Commun. 2016 Aug 23;4(1):89. doi: 10.1186/s40478-016-0359-7 (PMC4994254; doi:10.1186/s40478-016-0359-7)

Suppl. Figure 1

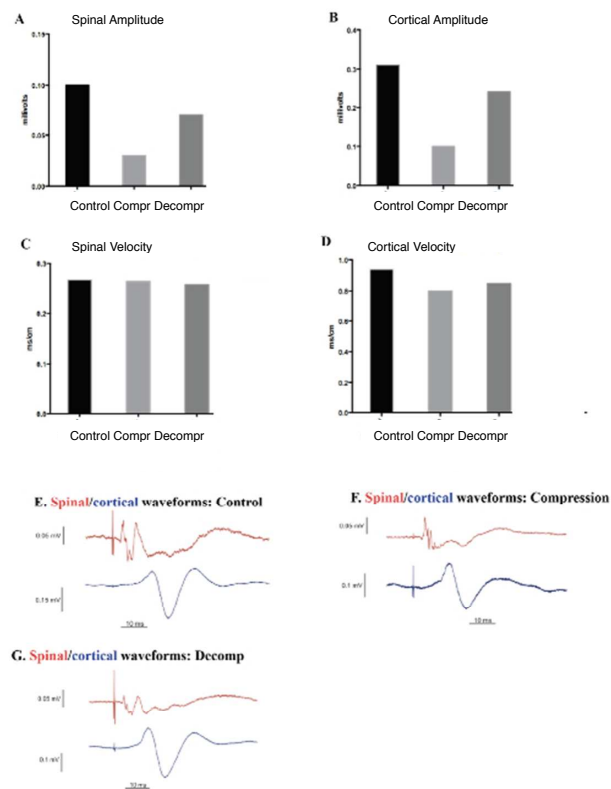

Supplement: Additional file 1: Figure S1. — Functional changes in the present model were reflected in sensory evoked potentials. For technical reasons only two rats were recorded in each group. Speed and amplitude of impulses transmitted from the tibial nerve in the lower limb to recording sites in the gracile nucleus of the lower brainstem (spinal) and the sensory cortex (cortical) were measured. Compression resulted in a decreased amplitudes of cortical and spinal waveforms. After decompressive surgery these increased but did not reach baseline amplitudes. (PDF 117 kb) [file 40478_2016_359_MOESM1_ESM.pdf]
